# Adaptive design – An innovative tool in drug development

**Published:** 2011-03

**Authors:** Ballari Brahmachari, Arun Bhatt

**Affiliations:** Medical & Regulatory Affairs Clininvent Research Pvt. Ltd, Mumbai, India bbrahmachari@clinivent.com; * Clininvent Research Pvt. Ltd., Mumbai, India *arunbhatt@ clininvent.com

The FDA in its 2004 Critical Path Initiative document highlighted an alarming decline in the number of innovative medicinal products being submitted for approval^1^. Huge cost and long-drawn time-frame associated with drug development, coupled with high rate of late phase attrition is recognized as primary reason for stagnation in today’s clinical development. Conventional clinical trials, with rigid designs often have low positive predictive value, implying, that these are inefficient in detecting ineffective drugs early in clinical development, leading to waste of time, money and resources. More flexible trial designs with efficient resource utilization are thus perceived as need of the hour^2^. Realizing this, both FDA and European Medicines Agency (EMA) have been encouraging what is known as adaptive trial design^2^,^3^. Recently, in 2010 February, the FDA released draft guidance for industry, detailing the prospects, challenges and requirements for adaptive design clinical trials^4^.

An adaptive design is defined as a clinical trial design that uses accumulating data to decide on how to modify aspects of the study as it continues, without undermining the validity and integrity of the trial^5^. Simply put, in an adaptive design, one attempts to learn from the accumulating data and then applies this learning in real-time to modify characteristics of ongoing trial. Trial aspects that could be modified include (but not restricted to) inclusion-exclusion criteria, treatment duration, dose, study end-point, evaluation criteria, randomization, study design, sample size, study hypothesis and statistical analysis plan. When compared to conventional designs, such flexibility (*i*) makes the study more efficient (fewer subjects, shorter duration), (*ii*) increases likelihood of success of study objective, and (*iii*) yields improved understanding of treatment effect (*e.g.*, better estimates of dose-response relationship or subgroup effects)^4^.

It needs to be emphasized that the use of adaptive design in a particular trial needs to be supported by a sound rationale. Such rationale might include treating trial patients as effectively and/or ethically as possible, minimizing overall drug development cost and making development process more efficient. This ensures that the most appropriate (i.e., ethical, efficient) means have been utilized in seeking answer to the research question. Adaptations are thus meant as a design feature to enhance trial efficiency and not as an *ad-hoc* rescue measure for poor planning^5^,^6^. In fact, study design revisions based on results of interim analyses that were not prospectively planned are not considered as adaptive design^4^.

Although the enthusiasm surrounding adaptive designs is relatively a recent phenomenon, concept-wise, the origin of adaptive designs can be traced back to 1970s when adaptive randomization and sequential trial designs were introduced^7^. Adaptive designs can be classified as prospective, concurrent or retrospective. Prospective adaptations (also known as design adaptations) imply that need for such adaptations are envisioned and approved in the protocol at the beginning of the trial. On the contrary, concurrent adaptations (also known as *ad-hoc* adaptation) imply changes that could not be envisioned at the beginning, but their need became apparent as trial continues. Retrospective adaptations generally imply changes in statistical analysis plan made prior to database lock or un-blinding^8^. Alternatively, adaptive designs can be categorized based on various rules that apply to the adaptation. Allocation rule defines how subjects will be allocated to different treatment arms (*e.g.*, response adaptive randomization); sampling rule defines how many subjects will be sampled in the next stage (*e.g.*, sample-size re-estimation); stopping rule defines when to stop the trial (*e.g.*, group sequential design); decision rule defines decisions pertaining to design change, not covered by the other three rules (*e.g.*, changes in hypothesis or study endpoint)^9^,^10^.

Examples of some commonly used adaptive designs include: (1) Adaptive randomization design wherein starting with equal number of subjects in each study arm, modification of randomization scheme is made based on treatment response and more patients are randomized to the arm showing better response, (2) Group sequential design that allows premature stopping of a trial based on efficacy / safety / futility, (3) Sample-size re-estimation that allows adjustment of sample size based on updated variance estimation at an interim look, (4) Drop-the-loser design which is a two-stage design mostly employed in phase II clinical development and allows dropping inferior treatment arm at the end of first stage and taking only the superior arm to the second stage, (5) Adaptive seamless phase II-III design having a learning stage (phase II) and a confirmatory stage (phase III) and the transition from phase II to phase III occurs without any gap, saving considerable time and using fewer number of subjects, (6) Adaptive dose finding design that is often used in early clinical development to identify the minimum effective dose and/or maximum tolerated dose, (7) Biomarker adaptive design that allows for adaptations based on response of biomarkers such as genomic markers, (8) Adaptive treatment switching design that allows switching of a patient’s treatment from an initial treatment to some alternative treatment, based on evidence of lack of efficacy or safety concerns of the initial treatment, (9) Adaptive hypothesis design that allows changes in hypothesis from superiority to non-inferiority before database lock or un-blinding, and (10) Multiple adaptive design which is a combination of any of the above designs^8^.

Despite their well-perceived advantages, the adaptive trials are complex to design, require more upfront planning, and are often met with statistical, procedural, logistic and regulatory challenges. Too many adaptations in the inclusion-exclusion criteria might result in incorporation of a totally different population, compared to what was originally planned. The major statistical concern arising out of too many such adaptations is that they tend to introduce operational bias and the overall type I error rate (i.e., erroneously claiming efficacy for an ineffective drug) might not be controlled^4^,^5^,^8^,^9^. This often calls for use of complicated Bayesian statistics along with more complex software programs to analyze adaptive trials. Similarly, too many changes in study endpoints might result in a different study altogether, unable to address the original research question^8^,^9^.

The crux of successful adaptation lies in preserving validity, integrity and quality of the trial, despite making changes based on interim analyses. To this end, it is imperative that all company staff involved in the conduct of trial remain blind to the results of interim analysis, so as to avoid any operational bias. Thus, setting up an Independent Data Monitoring Committee (IDMC) with responsibility to monitor accruing efficacy - safety data and recommend appropriate modifications is crucial for adaptive trials. The operation of such IDMC in adaptive trial is often challenging, as compared to its operation in a conventional trial. For example, sponsor perspectives might often be relevant to take the best decision regarding the trial. However, the extent of sponsor involvement in decision making process of IDMC needs fine tuning to ensure that independence of the IDMC is preserved^6^,^10^.

Procedural and logistic issues are another major challenge in implementation of adaptive trials^5^,^6^,^9^. First, the essence of adaptation is rapid collection of data, followed by prompt analysis in order to utilize the learning on a real-time basis. This means electronic data capture is a must in order to fully utilize the potential of an adaptive design. Second, an interactive communication system between investigators and sponsors is to be put in place to ensure that changes in study conduct are properly implemented. Third, since adaptive designs allow provision for changes in randomization scheme and/or sample-size re-estimation, management of drug supply and disposal needs to be carefully planned and requirement for any additional resources/ financial investments must be thought through before implementing adaptive trials. Fourth, most adaptive designs also require computer based trial-simulation to develop the design and protocol, implying need for sophisticated infrastructure^4^. Lastly, on a practical note, many Contract Research Organization/sponsor/Ethics Committee (EC) do not have long history or experience of monitoring an adaptive trial. Thus, proper training of trial staff and EC members becomes imperative.

Given that adaptive trials are liable to misuse (for example, *ad-hoc* changes to rescue a poorly planned study), regulators across the world often scrutinize adaptive designs much more closely. It is thus wise to enter into a discussion with the regulators early in the planning stage of adaptive studies, in order to understand what level of adaptation is acceptable and the overall regulatory expectations. Such expectations might include allowing only prospective adaptations for pivotal trials supporting product registration; clearly defined statistical methods to control type I error rate; written SOPs for adaptive design decisions; robust documentation practice; submission of trial simulation report and IDMC charter for regulatory review^6^,^8^,^10^. The advantage of such early regulatory communication is that, the regulatory medical or statistical expert reviewers can often offer valuable suggestions to improvise the trial based on data or models available within the agency.

To conclude, adaptive designs are not necessarily better than conventional designs in all trial settings. The merit of adaptive design over conventional design is to be judged on a case-by-case basis, looking at the overall clinical development plan. An adaptive design should be resorted to only when one is convinced of its merits over a conventional design. Further, implementation of adaptive trials is often fraught with challenges. Also, many areas of adaptive design still remain controversial and some regulatory agencies are still skeptical about acceptance of such designs. This explains why despite their attractiveness in terms of cost-efficiency and flexibility, adaptive designs are still in their infancy worldwide. Thus, while taking proactive measures towards implementation of adaptive designs regulators such as FDA are also leaving the industry with a word of caution: to allow time to build a better knowledge – base and understanding of adaptive designs before moving forward with their implementation^8^.

## References

[1] (2004). U.S. Department of Health and Human Services Food and Drug Administration. Innovation or Stagnation: Challenges and opportunity on the critical path to new medical products. USA.

[2] Woodcock J, Woosley R (2008). The FDA critical path initiative and its influence on new drug development. *Annu Rev Med*.

[3] (2006). European Medicines Agency. Reflection paper on methodological issues in confirmatory clinical trials with flexible design and analysis plan. London, UK.

[4] (2010). U.S. Department of Health and Human Services Food and Drug Administration. Guidance for industry – Adaptive design clinical trials for drugs and biologics. Draft Guidance USA.

[5] Gallo P, Chuang-Stein C, Dragalin V, Gaydos B, Krams M, Pinheiro J (2006). Phrma Working Group. Adaptive designs in clinical drug development– an Executive Summary of the PhRMA Working Group. *J Biopharm Stat*.

[6] Gaydos B, Anderson KM, Berry D, Burnham N, Chuang-Stein C, Dudinak J (2009). Good practices for adaptive clinical trials in pharmaceutical product development. *Drug Inf J*.

[7] Wei LJ (1978). The adaptive biased-coin design for sequential experiments. *Ann Stat*.

[8] Chow SC, Chang M (2008). Adaptive design methods in clinical trials – a review. *Orphanet J Rare Dis*.

[9] Mahajan R, Gupta K (2010). Adaptive design clinical trials: Methodology, challenges and prospect. *Indian J Pharmacol*.

[10] Aydemir U (2009). Requirements for adaptive designs in clinical trials. *Regulatory Focus*.

